# Methods and guidance on conducting, reporting, publishing and appraising living systematic reviews: a scoping review protocol

**DOI:** 10.12688/f1000research.55108.1

**Published:** 2021-08-13

**Authors:** Claire Iannizzi, Elie A Akl, Lara A Kahale, Elena Dorando, Abina Mosunmola Aminat, James M Barker, Joanne E. McKenzie, Neal R Haddaway, Vanessa Piechotta, Nicole Skoetz

**Affiliations:** 1 Evidence-based Oncology, Department I of Internal Medicine, Center for Integrated Oncology Aachen Bonn Cologne Duesseldorf, Faculty of Medicine and University Hospital Cologne, University of Cologne, Cologne, 50937, Germany; 2 Department of Medicine, American University of Beirut, Beirut, Lebanon; 3 Department of Health Research Methods, Evidence, and Impact, McMaster University, Hamilton, Canada; 4 Editorial and Methods Department, Cochrane Central Executive, Cochrane, London, SW1Y 4QX, UK; 5 Rafic Hariri School of Nursing, American University of Beirut, Beirut, Lebanon; 6 F1000 Research Ltd, London, SE1 8BU, UK; 7 School of Public Health and Preventive Medicine, Monash University, Melbourne, Victoria, Australia; 8 Stockholm Environment Institute, Stockholm, Sweden; 9 Mercator Research Institute on Global Commons and Climate Change, Berlin, Germany; 10 Africa Centre for Evidence, University of Johannesburg, Johannesburg, South Africa

**Keywords:** Living systematic reviews, methods and guidance, scoping review, conducting LSRs, reporting, appraisal

## Abstract

**Background: **The living systematic review (LSR) approach is based on an ongoing surveillance of the literature and continual updating. A few guidance documents address the conduct, reporting, publishing and appraisal of systematic reviews (SRs), but the methodology described is either not up-to date or not suitable for LSRs and misses additional LSR-specific considerations. The objective of this scoping review is to systematically collate methodological literature and guidance on how to conduct, report, publish and appraise the quality of LSRs. The scoping review will allow the mapping of the existing evidence on the topic to support LSRs authors seeking guidance and identify related gaps.

**Methods: **To achieve our objectives, we will conduct a scoping review to survey and evaluate existing evidence, using the standard scoping review methodology. We will search MEDLINE, EMBASE, and Cochrane using the OVID interface. The search strategy was developed by a researcher experienced in developing literature search strategies with the help of an information specialist. As for searching grey literature, we will seek existing guidelines and handbooks on LSRs from organizations that conduct evidence syntheses using the Lens.org website. Two review authors will extract and catalogue the study data on LSR methodological aspects into a standardized and pilot-tested data extraction form. The main categories will reflect proposed methods for (i) conducting LSRs, (ii) reporting of LSRs, (iii) publishing and (iv) appraising the quality of LSRs.

**Data synthesis and conclusion: **By collecting these data from methodological surveys and papers, as well as existing guidance documents and handbooks on LSRs, we might identify specific issues and components lacking within current LSR methodology. Thus, the systematically obtained findings of the scoping review could be used as basis for the revision of existing methods tools on LSR, for instance a PRISMA statement extension for LSRs.

## Background

Systematic reviews (SRs) are essential to provide evidence-based answers to clinical and public health-related questions. Due to continuous publishing of relevant primary studies in some areas, it is important to keep these SRs up-to-date. ^1^ One could achieve that goal by adopting the living systematic review (LSR) approach, which is based on an ongoing surveillance of the literature and continual updating. ^2^ Regular searches ensure that the SR includes the latest available findings and remains up-to-date. ^2^ Therefore, LSRs are most suitable for high-priority topics with substantial uncertainty and frequent publications.When continually updating a review, it is important but challenging to report changes to the methodology and to the findings in transparent and traceable ways.

Few guidance documents address the conduct, reporting, publishing and appraisal of LSRs. The Living Evidence Network developed the “Guidance for the production and publication of Cochrane living systematic reviews”, but that guidance is now four years old. ^3^ While the recent update of the ‘Preferred Reporting Items for Systematic reviews and Meta-Analyses’ (PRISMA) can be used for reporting living systematic reviews, the statement indicates there may be some additional considerations that need to be addressed. ^4^ On the other hand, the AMSTAR - Assessing the Methodological Quality of Systematic Reviews tool which was developed for the critical appraisal of the quality of SRs, does not consider LSRs. ^5^

Therefore, it is of high interest to summarize the literature evaluating methods of conducting, reporting, publishing and appraising LSRs, as well as any guidance on those methods.

## Objective

The objective of this scoping review is to systematically collate methodological literature and guidance on how to conduct, report, publish and appraise the quality of LSRs. The scoping review will allow the mapping of the existing evidence on the topic to support LSRs authors seeking guidance. Also, the scoping review will identify the knowledge gaps in the field and any need to revise existing guidance or develop new ones for conducting, reporting and appraising LSRs.

## Methods

### Overview and definitions

**Figure 1.**
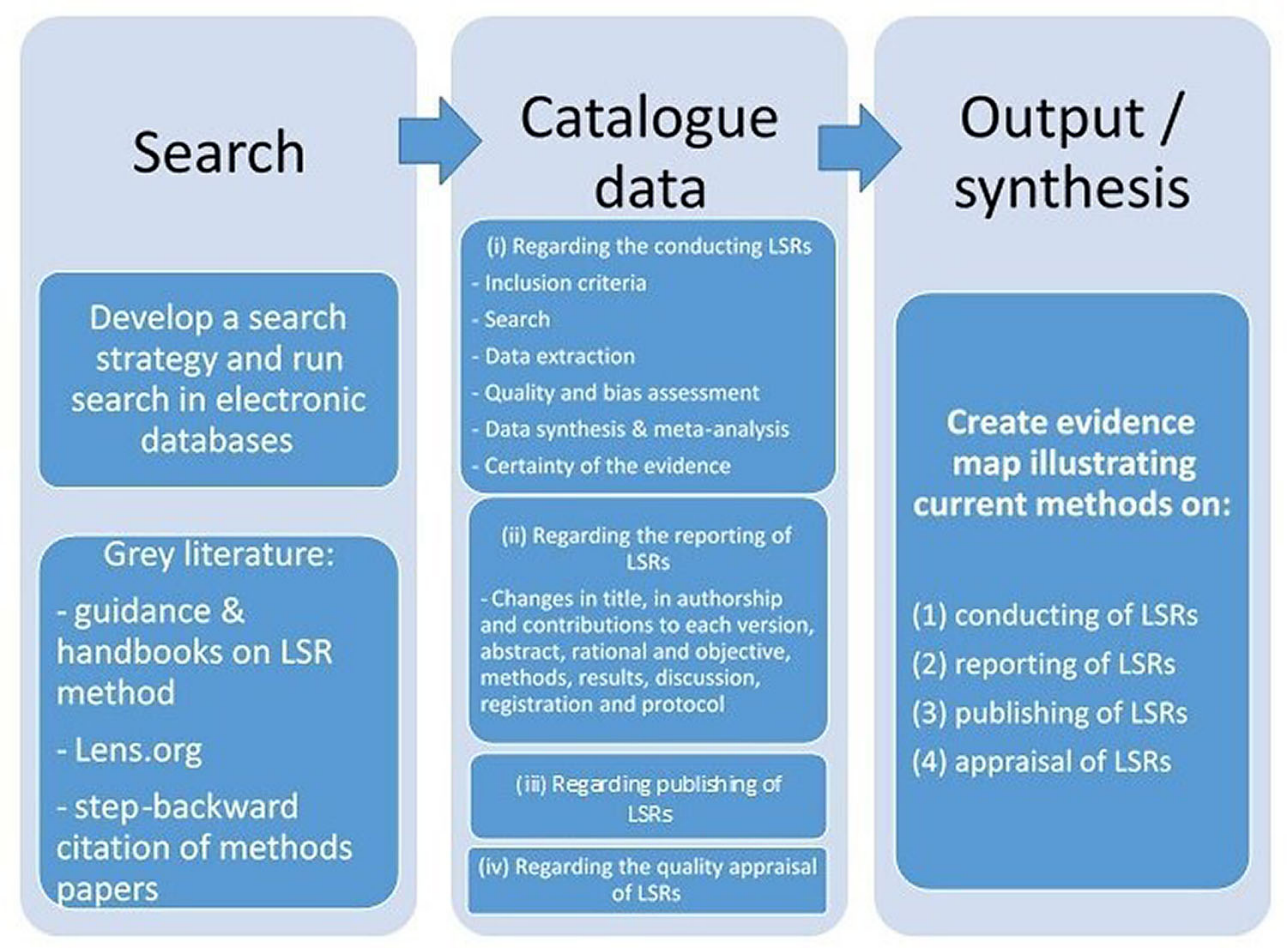
Framework on the methodological plan, from search to data synthesis, for this scoping review.

This scoping review study will be part of a larger project to develop an extension of the PRISMA 2020 statement for living systematic reviews. To achieve our objectives, we will conduct a scoping review to survey and evaluate existing evidence and literature, especially with regards to the availability of methods papers, evidence gaps and associated primary research gaps. ^6^ A framework is illustrating an overview of the methodological plan for this scoping review from the search to the data synthesis ( Figure 1). Scoping reviews are particularly useful in the context of emerging evidence and act as a precursor for other topic-related projects. ^6^ We will use standard scoping review methodology with the following steps:

a) identification of the research question;
b) identification of relevant studies;
c) study selection;
d) charting the data; and
e) collating, summarizing and reporting of the results. ^7^

### Eligibility criteria

We will include articles that devoted at least two paragraphs to discuss methods or conceptual approaches for how to conduct, report, publish or appraise LSRs. Such articles should ideally include methodological or concept papers describing methods for LSRs, guidance ( *e.g.* handbooks) for undertaking LSRs, issued by organizations that conduct evidence syntheses, and commentaries or editorials that discuss methods for LSR.

We will exclude from our search, LSRs themselves, as well as LSR protocols.

### Search methods for identification of studies

We will search in MEDLINE, EMBASE, and Cochrane from 2013 using the OVID interface. The search strategy was developed by a researcher experienced in developing literature search strategies with the help of an information specialist (LH) as part of a larger project to develop an extension of the PRISMA 2020 statement for LSRs. ^8, 9^ The strategy was peer-reviewed by an information specialist (IM) who will also run the search planned for July or August 2021. Please see Box 1 for the complete search strategy.

Box 1. **Study search strategy**

Medline

1. exp Meta-Analysis as topic/
2. Systematic Reviews as Topic/
3. (meta-analysis or review or systematic review).pt. or search*.tw.
4. 1 or 2 or 3
5. ((continuous* or continual* or continue or periodic*) adj3 (updat* or search*)).mp.
6. 4 and 5
7. ((living adj3 (review* or metaanaly* or (meta adj analy*))) or lsr).mp.
8. 6 or 7
9. limit 8 to yr="2013 -Current"

Embase

1. 'meta analysis'/exp OR 'meta analysis (topic)'/de
2. 'systematic review'/de OR 'systematic review(topic)'/de
3. 'meta analys$s':ti OR review*:ti OR metanalys$s:ti OR search:ti,ab
4. #1 OR #2 OR #3
5. ((continuous* OR continual* OR continue OR periodic*) NEAR/3 (updat* OR search*)):ti,ab,kw
6. #4 AND #5
7. ((living NEAR/3 (review* OR metaanaly* OR 'meta analy*' OR metanaly*)):ti,ab,kw) OR lsr:ti,ab,kw
8. #6 OR #7
9. #8 AND (2013:py OR 2014:py OR 2015:py OR 2016:py OR 2017:py OR 2018:py OR 2019:py OR 2020:py OR 2021:py)

Cochrane:

1. MeSH descriptor: [Meta-Analysis] this term only
2. MeSH descriptor: [Meta-Analysis as Topic] explode all trees
3. MeSH descriptor: [Systematic Review] this term only
4. MeSH descriptor: [Systematic Reviews as Topic] this term only
5. ((((meta-analys?s or review* or metanalys?s or metaanalys?s)))):pt
6. (((search*))):ti OR (((search*))):ab
7. #1 OR #2 OR #3 OR #4 OR #5 OR #6
8. ((((continuous* OR continual* OR continue OR periodic*) NEAR/2 (updat* or search*)))):ti,ab,kw
9. #7 AND #8
10. (((living NEAR/2 (evidence OR metaanaly* OR meta-analy* OR metanaly*))) OR LSR):ti,ab,kw
  #9 OR #10 with Cochrane Library publication date Between Jan 2013 and April 2021

As for searching the “grey literature”, we will seek existing guidelines and handbooks on LSRs from organizations that conduct evidence syntheses ( *e.g.* Cochrane handbook, Living Evidence network, JBI) using the Lens.org website. Additionally, we will conduct a step-backwards citation approach to identify relevant LSRs handbooks and guidance documents from the reference list of published LSRs. We will also use forward searching, using certain seminal documents ( *e.g.* papers defining LSRs and Cochrane guidance) and track their citations *via* google scholar.

### Article selection

Two authors (CI, AM) will independently and in duplicate screen titles and abstracts. We will use a web-based systematic review software Rayyan (RRID:SCR_017584) for the screening process. To ensure a consistent screening procedure and optimize agreement, we will develop and use a detailed written instruction form. We will then screen for full text assessing eligibility, based on our predefined eligibility criteria. Disagreements and conflicts will be solved by a third author.

### Data collection and charting

Two review authors will extract and catalogue the study data on LSR methodological aspects into a standardized and pilot tested data extraction form in Microsoft Excel (RRID:SCR_016137); an open-access alternative is Google Sheets (RRID:SCR_017679). All reviewers will participate in calibration exercises before data abstraction to improve reliability, and the senior investigator will serve as a third independent reviewer for resolving disagreements. The predefined categories of data extraction will be piloted, and we will chart data regarding proposed methods for (i) conducting LSRs, (ii) reporting of LSRs, (iii) publishing and (iv) appraising the quality of LSRs. Even though we extract and classify the data according to these categories, we consider that elements from one category ( *e.g.* conducting LSR) can have an impact on elements from another category ( *e.g.* publishing LSR) and might even overlap. We will specifically extract data on the following items:

(i) Regarding the conduct of LSRs
  • Inclusion criteria (change, re-evaluation)
  • Search (frequency, when, database)
  • Data extraction (who, frequency, tool)
  • Quality and bias assessment of identified studies (who, frequency, tool)
  • Data synthesis with meta-analysis if applicable (who, frequency, tool)
  • Certainty of the evidence (who, frequency, tool)
(ii) Regarding the reporting of LSRs:
  • Title (identified as LSR)
  • Methods
  • Results
  • Discussion
  • Registration and protocol (New protocol for each version with or without changes)
  • Support
  • Funding and competing interests
  • Availability of data
(iii) Regarding the publication of LSRs:
  • Publication types of new findings (full revised manuscript or letter)
  • Communicating review status
  • New citation (visibility)
  • Publication of an update/decision on update (frequency, when, trigger)
  • Between updates
  • Transition out of living mode (when, trigger)
  • Peer review
(iv) Regarding the appraisal of LSRs:
  • PICO structure applied
  • Methods established prior to the conduct and justification of protocol deviations
    ⁰ Difference between protocol and review assessed
    ⁰ Difference between review versions assessed
  • Study selection explained
  • Use of comprehensive search strategy
  • Study registry search
  • Ongoing studies search
  • Study selection and data extraction performed in duplicate
  • List of excluded studies with justification
  • Description of included studies
  • Adequate tool for risk of bias assessment
  • Appropriate methods for meta-analysis
  • Usage/handling of preprints
  • Funding sources, COI

### Data synthesis

We will collect, summarize and report the extracted study data according to the predefined categories. Therefore, we will first be presenting a table with the extracted methods unique to LSRs classified into the pre-defined categories, along with the contributing sources. Second, we will develop a visual evidence map framework of these methods and map our findings against the existing standard tools ( *e.g.* Cochrane guidance for conducting LSR, PRISMA 2020, AMSTAR 2) to determine differences and suggestions for adaptations. The evidence map and the summary table will be double-checked by a second review author. These systematically obtained findings of the scoping review could be used as basis for the revision of existing guidance and method tools for LSR, such as the PRISMA statement for instance.

### Dissemination of information

We plan to disseminate the findings by submitting the resulting scoping review for publication to F1000Research.

### Study status

We are currently developing and piloting the data extraction form and the next step will be to run the search and start screening.

## Data availability

### Underlying data

No data are associated with this article.
