# Supplementary material for: Methods and guidance on conducting, reporting, publishing and appraising living systematic reviews: a scoping review protocol
Source: F1000Res. 2021 Aug 13;10:802. [Version 1] doi: 10.12688/f1000research.55108.1 (PMC8822136; doi:10.12688/f1000research.55108.1)
Supplement: Peer review history

# Peer review: Reviewer response for version 1

**DOI:** 10.5256/f1000research.58654.r98640

**Published:** 2022-02-07

**Authors:** Lyndsay A. Alexander

**Affiliations:** 1 The Scottish Centre for Evidence-based, Multi-professional Practice: A JBI Centre of Excellence, Aberdeen, UK; 2 School of Health Sciences, Robert Gordon University, Aberdeen, UK

Thank you for the invitation to review this scoping review protocol. This is an interesting and well presented protocol on a timely topic.

 I do not have many comments on this manuscript:

- I would be interested in why the authors cite Arksey & O'Malley as the scoping review methodology when there is more up to date and expanded scoping review methodology available (e.g. JBI Scoping review methodology- Peters *et al.* 2020).
- Why was 2013 chosen as the starting search date?
- For the grey literature search - what are the amended search terms that will be used? From the manuscript, I would not be able to replicate this aspect of the search as currently presented.

Is the study design appropriate for the research question?

Yes

Is the rationale for, and objectives of, the study clearly described?

Yes

Are sufficient details of the methods provided to allow replication by others?

Partly

Are the datasets clearly presented in a useable and accessible format?

Not applicable

Reviewer Expertise:

Member of JBI Scoping review methodology group.

I confirm that I have read this submission and believe that I have an appropriate level of expertise to confirm that it is of an acceptable scientific standard.

## References

[1] : Updated methodological guidance for the conduct of scoping reviews. *JBI Evid Synth* .18(10) : 10.11124/JBIES-20-00167 2119-2126 10.11124/JBIES-20-00167 33038124

# Peer review: Reviewer response for version 1

**DOI:** 10.5256/f1000research.58654.r96319

**Published:** 2021-10-19

**Authors:** Zachary Munn

**Affiliations:** 1 Transfer Science Department, Faculty of Health and Medical Sciences, Joanna Briggs Institute, The University of Adelaide, Adelaide, SA, Australia

Thank you to the editors for providing the opportunity to peer review this scoping review protocol investigating methods and guidance on conducting, reporting, publishing and appraising living systematic reviews. The choice of a scoping review approach is methodologically sound and it is encouraging to see the authors have planned their scoping review a priori with this protocol. This is important and interesting work and I commend the authors for taking on this project. I have relatively few minor comments below, which are all at the author’s discretion and none are essential as the article is methodologically sound in its current form. 

 Minor comments:

- It may be worth mentioning the final scoping review will be reported in line with PRISMA ScR.
- For your eligibility criteria, it would be useful to know if you are including all living review guidance, regardless of discipline/field.
- Justify the 2013 search date (i.e. living reviews are a recent innovation…)
- How will the visual evidence map be created? If using software, it would be good to mention this.
- What contribution will this study provide in addition to the protocol/planned study by Khamis *et al.*? It might be worth mentioning this in the background.
- Typos/copyediting
- "A framework is illustrating an overview of the methodological plan for this scoping review from the search to the data synthesis." – consider rephrasing.

Is the study design appropriate for the research question?

Yes

Is the rationale for, and objectives of, the study clearly described?

Yes

Are sufficient details of the methods provided to allow replication by others?

Yes

Are the datasets clearly presented in a useable and accessible format?

Not applicable

Reviewer Expertise:

Systematic reviews, guidelines, evidence synthesis

I confirm that I have read this submission and believe that I have an appropriate level of expertise to confirm that it is of an acceptable scientific standard.

# Response

**Published:** 2021-12-14

**Authors:** Claire Iannizzi

**Affiliations:** University Hospital of Cologne, Germany

Dear Reviewer, 

 Thank you very much for your review and comments on our protocol. We will incorporate your suggested edits for our final scoping review. We will mention the reporting of the final scoping review in line with PRISMA ScR, the eligibility criteria will be slightly specified, we will elaborate more on the evidence map and the background.
